# Outcomes of Convalescent Plasma with Defined High versus Lower Neutralizing Antibody Titers against SARS-CoV-2 among Hospitalized Patients: CoronaVirus Inactivating Plasma (CoVIP) Study

**DOI:** 10.1128/mbio.01751-22

**Published:** 2022-09-22

**Authors:** Luther A. Bartelt, Alena J. Markmann, Bridget Nelson, Jessica Keys, Heather Root, Heather I. Henderson, JoAnn Kuruc, Caroline Baker, D. Ryan Bhowmik, Yixuan J. Hou, Lakshmanane Premkumar, Caleb Cornaby, John L. Schmitz, Susan Weiss, Yara Park, Ralph Baric, Aravinda M. de Silva, Anne Lachiewicz, Sonia Napravnik, David van Duin, David M. Margolis

**Affiliations:** a Department of Medicine, Division of Infectious Diseases, University of North Carolina School of Medicine, Chapel Hill, North Carolina, USA; b Department of Microbiology and Immunology, University of North Carolina School of Medicine, Chapel Hill, North Carolina, USA; c The AIDS Center at Montefiore, Division of Infectious Diseases, Montefiore Medical Center, Bronx, New York, USA; d UNC HIV Cure Center, University of North Carolina School of Medicine, Chapel Hill, North Carolina, USA; e Department of Epidemiology, University of North Carolina at Chapel Hill, Chapel Hill, North Carolina, USA; f Department of Pathology & Laboratory Medicine, University of North Carolina School of Medicine, Chapel Hill, North Carolina, USA; g Department of Pathology, Carolinas Pathology Group, Atrium Health Carolinas Medical Center, Charlotte, North Carolina, USA; Johns Hopkins Bloomberg School of Public Health

**Keywords:** antibodies, SARS-CoV-2, convalescent plasma, immunology, neutralizing antibodies, antibodies, coronavirus, immunology

## Abstract

COVID-19 convalescent plasma (CCP) was an early and widely adopted putative therapy for severe COVID-19. Results from randomized control trials and observational studies have failed to demonstrate a clear therapeutic role for CCP for severe acute respiratory syndrome coronavirus 2 (SARS-CoV-2) infection. Underlying these inconclusive findings is a broad heterogeneity in the concentrations of neutralizing antibodies (nAbs) between different CCP donors. We conducted this study to evaluate the effectiveness and safety of nAb titer-defined CCP in adults admitted to an academic referral hospital. Patients positive by a SARS-CoV-2 nucleic acid amplification test and with symptoms for <10 days were eligible. Participants received either CCP with nAb titers of >1:640 (high-titer group) or ≥1:160 to 1:640 (standard-titer group) in addition to standard of care treatments. The primary clinical outcome was time to hospital discharge, with mortality and respiratory support evaluated as secondary outcomes. Adverse events were contrasted by CCP titer. Between 28 August and 4 December 2020, 316 participants were screened, and 55 received CCP, with 14 and 41 receiving high- versus standard-titer CCP, respectively. Time to hospital discharge was shorter among participants receiving high- versus standard-titer CCP, accounting for death as a competing event (hazard ratio, 1.94; 95% confidence interval [CI], 1.05 to 3.58; Gray’s *P* = 0.02). Severe adverse events (SAEs) (≥grade 3) occurred in 4 (29%) and 23 (56%) of participants receiving the high versus standard titer, respectively, by day 28 (risk ratio, 0.51; 95% CI, 0.21 to 1.22; Fisher’s *P* = 0.12). There were no observed treatment-related AEs. (This study has been registered at ClinicalTrials.gov under registration no. NCT04524507).

## INTRODUCTION

COVID-19 convalescent plasma (CCP) was one of the first putative therapies to become widely adopted after the emergence of the novel virus severe acute respiratory syndrome coronavirus 2 (SARS-CoV-2). Since the start of the pandemic and FDA authorization for inpatient use in September 2020, CCP has been infused to >500,000 hospitalized patients in the United States (1). However, data from randomized control trials (RCTs) comparing CCP to the standard of care or CCP to plasma devoid of anti-SARS-CoV-2 antibodies remain mixed. Reported benefits in accelerated recovery and decreased mortality in some CCP RCTs (2) have not been observed in the largest RCTs (3–5). Underlying the challenges in conducting and interpreting CCP clinical trials are heterogeneity in the source of the CCP product and wide interindividual variability in the breadth and potency of anti-SARS-CoV-2 neutralizing antibodies (nAbs) and antibodies with nonneutralizing functions between different CCP donors (6). Indeed, secondary analyses in the CONCOR-1 trial (ClinicalTrials registration no. NCT04348656) identified that antibody content and/or CCP supplier had a significant effect on estimates of 30-day mortality and the need for ventilation among the CCP-treated participants (4). Findings in large multicenter community-based observational studies suggest that CCP with higher anti-SARS-CoV2 antibody content than CCP with lower anti-SARS-CoV2 antibody by OrthroVitros enzyme-linked immunosorbent assay (ELISA) testing is associated with a lower risk of in-hospital mortality (7–9), and more recently, use of high-antibody-titer CCP in outpatients reduced risk for hospitalization (10). These findings led to Emergency Use Authorization from the FDA and recommendations from expert societies to use high-antibody-titer CCP as an outpatient therapeutic for high-risk individuals when other therapies are not available (11).

Antibody content is considered a major determinant of CCP safety and effectiveness; however, there remains no standardized assay for distinguishing high-antibody-titer CCP with potential clinical benefit from CCP without benefit or even CCP that some have proposed may be harmful (4). For example, the FDA initially recommended neutralizing antibody (nAb) titers of >1:160 as qualification for CCP as treatment. However, the limited ability to perform live viral neutralization assays prohibited most trials from using this threshold. Rather, large trials like RECOVERY (ClinicalTrials registration no. NCT04381936) qualified high-titer CCP using an anti-SARS-CoV-2 spike (S) protein IgG antibody ELISA (EUROIMMUN) with an index value of >6.0 (3). In contrast, CCP in the U.S. Expanded Access Program (EAP) observational study in 30,000 patients used the OrthoVitros ELISA platform to quantify anti-SARS-CoV-2 antibody content to assign high-antibody and lower-antibody CCP (7). While these spike protein-targeted ELISAs correlate with neutralizing antibody titers, we and others have shown that spike protein IgG titers, including IgG titers targeted to the spike protein receptor binding domain (RBD), correlate, but alone they are imperfect surrogates for precise functional viral neutralizing capability of CCP (12, 13).

To circumvent the limitation of relying on a single antibody-binding assay as a measure of the global antiviral activity of a polyclonal therapy like CCP, we designed a study administering CCP with predefined ranges of neutralizing antibody. Using an on-site SARS-CoV-2-WA1 viral reporter neutralization assay also used in the development of mRNA vaccines, we precisely defined the functional viral inhibitory properties of CCP. Beginning in April 2020, our medical center began collecting locally sourced CCP for use in our hospital (6). Here, we report outcomes from a study in patients admitted with severe COVID-19 using pretreatment nAb titer-defined CCP from our donor cohort in two different ranges: >1:640 (high titer) and the FDA minimum recommended nAb titer of 1:160 to 1:640 (standard titer). The primary effectiveness outcome was time to hospital discharge, and the primary safety outcome was adverse events (AEs) attributable to CCP.

## RESULTS

**FIG 1:**
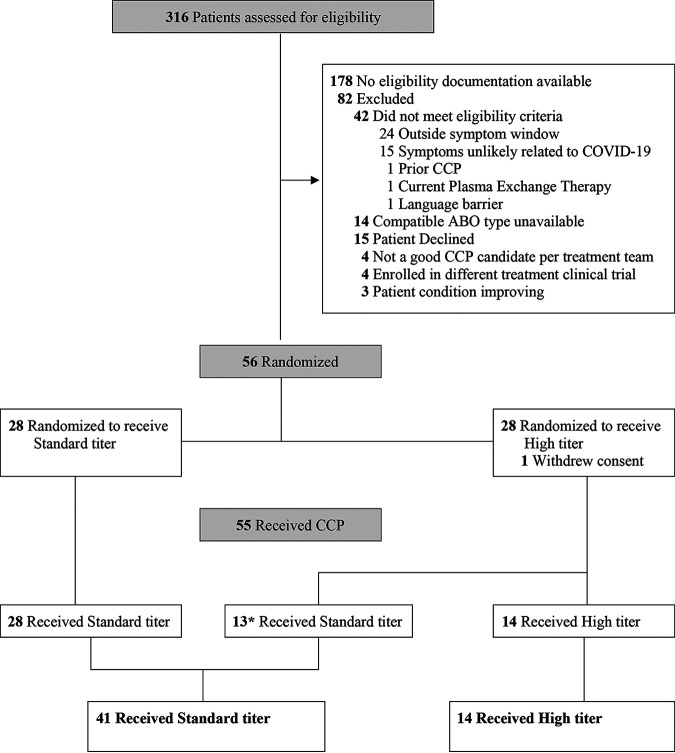
Enrollment, randomization, and treatment allocation. Patient flow diagram in the CoVIP study, detailing excluded patients, randomization, and CCP titer received. *, 13 patients randomized to receive high-titer CCP received standard-titer CPP because ABO-compatible high-titer plasma was not available.

Between 28 August and 4 December 2020, 316 patients were assessed for eligibility and 55 received CCP: 14 and 41 participants received high- and standard-titer CCP, respectively (Fig. 1). All but one participant received 2 full U of CCP (54 of 55 patients), with one participant receiving the standard titer completing only the first unit. The 2 U of donor-identical CCP (200 to 300 mL each) was administered on the day of randomization (baseline), a median of 5.1 h apart (interquartile range [IQR], 4.4 to 7.3; full range, 0.9 to 28.7). Overall, patients were a median of 61 years of age (IQR, 52 to 67), 36% were female, 25% Black, and 33% Hispanic. Demographic and clinical patient characteristics at baseline were comparable by CCP titer received (Table 1), including respiratory support requirements and the administration of other pharmacological COVID-19-directed therapies.

**TABLE 1:** Baseline demographic and clinical characteristics by nAb titer of CCP received^*a*^

Parameter	Result for nAb titer of CCP		*P* value
	High (*n* = 14)	Standard (*n* = 41)	
Age, median yr (IQR)	56 (49–75)	62 (54–67)	0.68
Female, no. (%)	6 (43)	14 (34)	0.75
Race/ethnicity, no. (%)			0.50
White	7 (50)	14 (34)	
Black	2 (14)	12 (29)	
Hispanic	5 (36)	13 (32)	
Other	0	2 (5)	
Blood type, no. (%)			0.53
A	5 (36)	16 (39)	
AB	0	1 (2)	
B	0	4 (10)	
O	9 (64)	20 (49)	
Coexisting condition(s) and medication(s), median no. (%)			
Any^*b*^	13 (93)	36 (88)	0.99
Hypertension	1 (7)	10 (24)	0.25
Diabetes	5 (36)	19 (46)	0.54
Obesity	9 (64)	23 (56)	0.76
Cardiovascular disease	2 (14)	5 (12)	0.99
Chronic pulmonary disease	0 (0)	9 (22)	0.09
Solid tumor	0 (0)	3 (7)	0.56
Hematologic malignancies	0 (0)	2 (5)	0.99
Solid organ transplant	1 (7)	11 (27)	0.16
Hematologic stem cell transplantation	0 (0)	1 (2)	0.99
Immunosuppressive medication	2 (14)	14 (34)	0.19
Days from symptom onset, median no. (IQR)	6 (5–8)	7 (3–8)	0.61
Days from hospital admission, median no. (IQR)	1 (1–2)	2 (1–2)	0.47
WHO clinical status, median no. (IQR)^*c*^	6 (4–6)	6 (5–6)	0.89
Respiratory support, no. (%)			0.63
None	2 (14)	6 (15)	
LFNC	5 (36)	13 (32)	
Noninvasive ventilation	5 (36)	20 (49)	
Invasive ventilation	2 (14)	2 (5)	
COVID-19 medication, no. (%)^*d*^			
Remdesivir	12 (86)	39 (95)	0.27
Glucocorticoid	13 (93)	37 (90)	0.99
Anticoagulant	13 (93)	39 (95)	0.99
Antibacterial agent	7 (50)	28 (68)	0.33
Anti-SARS-CoV-2 nAb titer^*e*^	1:26 (ND–1:49)	1:24 (ND–1:76)	0.98

a ND, not detected; LFNC, low-flow nasal cannula. *P* values were calculated using Fisher’s exact test, Pearson’s χ^2^ test, or Wilcoxon rank-sum test, as indicated in the text.

b Any coexisting condition includes any of the conditions listed.

c WHO ordinal scale with categories: 4 indicates hospitalized with oxygen by mask or nasal prongs, 5 indicates hospitalized with noninvasive ventilation or high-flow oxygen, and 6 indicates hospitalized with oxygen by noninvasive ventilation or high flow.

d COVID-19 medications used at or prior to baseline.

e Median anti-SARS-CoV-2 nAb titer and IQR available for *n* = 7 in the high-titer CCP group and *n* = 27 in the standard-titer CCP group.

### Clinical outcomes.

**FIG 2:**
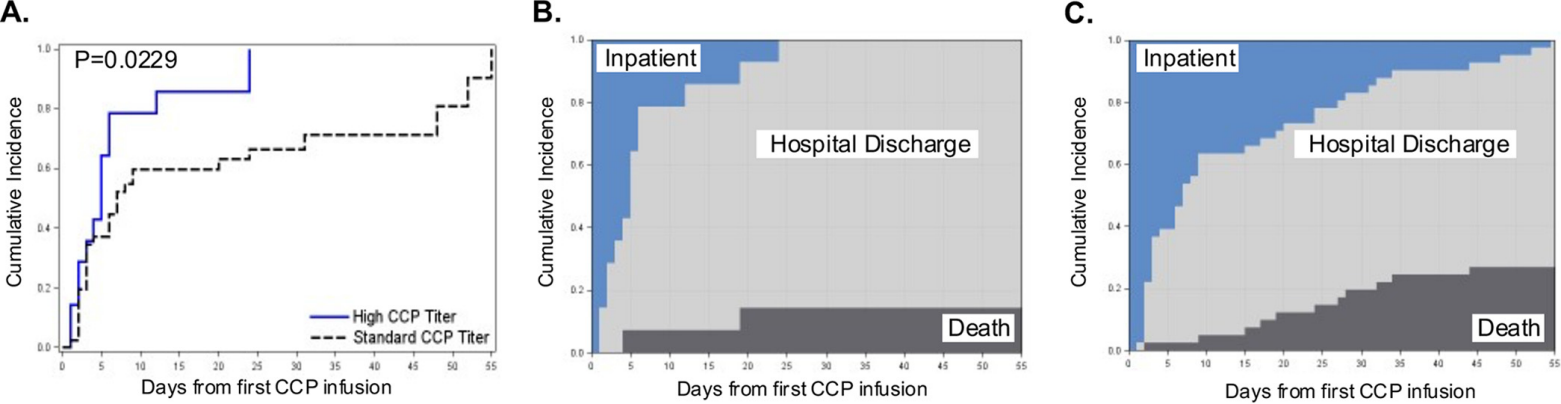
Cumulative incidence of hospital discharge by high- versus standard-neutralizing antibody-titer CCP received. Time to hospital discharge from first CCP infusion until day 55, by CCP titer received with death as a competing event, was estimated using the Aalen-Johansen estimator with Gray’s test with rho = 0 (A). Shown are stacked cumulative incidence curves for death, hospital discharge, and remaining hospitalized as competing risks among patients receiving high-titer CCP (B) and standard-titer CCP (C). Deaths are shaded in black, hospital discharge in gray, and remaining hospitalized in blue.

**FIG 3:**
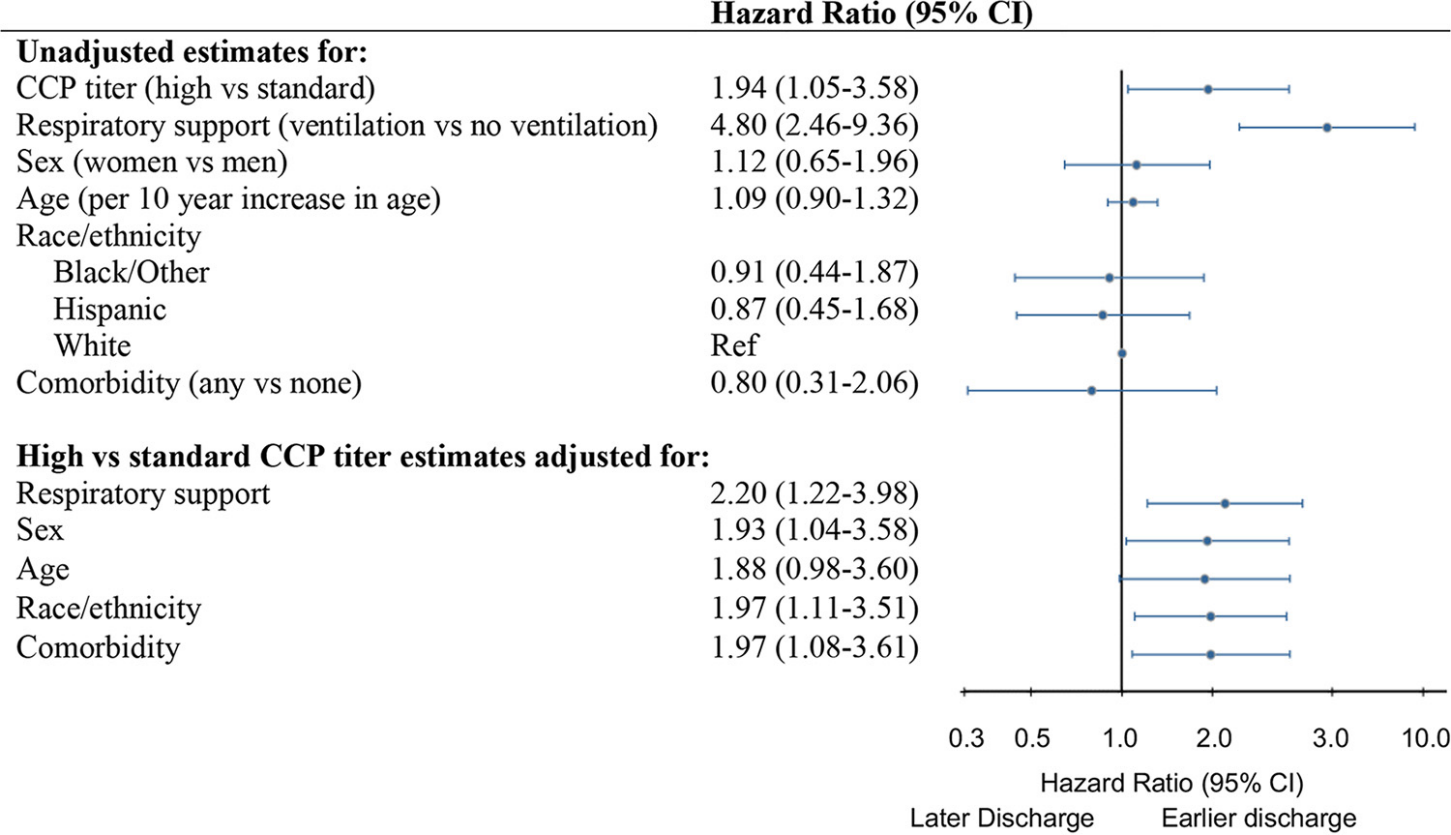
Time to hospital discharge by neutralizing antibody CCP titer received and patient characteristics. Hazard ratios and 95% confidence intervals for hospital discharge from first CCP infusion until day 55 were estimated by the Fine-Gray method, accounting for competing risk of death. Shown are unadjusted estimates for high- versus standard-titer CCP and select patient characteristics fit with separate models. High- versus standard-titer CCP adjusted estimates with adjustment made for each patient characteristic are given in a separate model. Respiratory support measured at baseline contrast is ventilation (i.e., noninvasive or invasive ventilation) versus less than ventilation (i.e., none or low-flow nasal cannula). Comorbidities (any) included diagnosis at baseline of any of the following: hypertension, diabetes, obesity, cardiovascular disease, chronic pulmonary disease, solid tumor, hematologic malignancies, solid organ transplant, and hematologic stem cell transplantation.

Patients receiving high- versus standard-titer CCP in the primary as-treated analyses had a shorter time to hospital discharge from baseline to day 55, accounting for the competing risk of death (Gray’s *P* = 0.0229) (Fig. 2A). Differences in the cumulative incidence curves for death and hospital discharge favored patients receiving high- versus standard-titer CCP across follow-up from baseline through day 55 (Fig. 2B and C). The unadjusted hazard ratio (HR) of time to hospital discharge from baseline through day 55 also favored patients receiving high- versus standard-titer CCP, with patients receiving a high titer having a shorter time to hospital discharge than those receiving the standard titer, with a hazard ratio contrasting patients receiving a high versus standard titer of 1.94 (95% confidence interval [CI], 1.05 to 3.58) (Fig. 3).

Assessing for confounding of the hazard ratio for time to hospital discharge by titer received, we did not observe any association between any of our possible confounders and the titer participants received, including age, sex, race/ethnicity, respiratory support needs at enrollment, and presence of any comorbidity (hypertension, diabetes, obesity, cardiovascular disease, chronic pulmonary disease, solid tumor, hematologic malignancies, solid organ transplant, and hematologic stem cell transplantation) (Table 1). Not requiring either noninvasive or invasive ventilation at baseline was strongly associated with a shorter time to hospital discharge (hazard ratio, 4.80; 95% CI, 2.46 to 9.36) (Fig. 3). Other patient characteristics were not associated with hospital discharge in this study population. The adjusted hazard ratios for time to hospital discharge by titer received were consistent with the primary unadjusted findings. For example, after adjusting for respiratory support, the adjusted hazard ratio favored patients receiving high- versus standard-titer CCP (adjusted hazard ratio, 2.20; 95% CI, 1.22 to 3.98).

In secondary outcomes, patients receiving high- versus standard-titer CCP had a shorter time to hospital discharge from baseline through day 14 and day 28, consistent with day 55 results (Table 2). Through day 55 postbaseline, fewer patients receiving high- versus standard-titer CCP died (14 versus 27%; *P* = 0.27); however, mortality differences were not statistically significantly different given the imprecision in the estimates. No statistically significant differences in days of respiratory support and WHO clinical status were observed by CCP titer received.

**TABLE 2:** Secondary outcomes by nAb titer of CCP received^*a*^

Parameter	Result for nAb titer of CCP		*P* value
	High (*n* = 14)	Standard (*n* = 41)	
Hospital discharge^*b*^			
Day 14 HR (95% CI)	1.96 (1.05–3.67)	1.0	
Day 28 HR (95% CI)	2.06 (1.15–3.68)	1.0	
Mortality^*c*^			
Day 14 deaths, *n* (%)	0 (0)	2 (7)	0.41^*c*^
Day 14 HR (95% CI)	NA^*d*^		
Day 28 deaths, *n* (%)	2 (14)	8 (20)	0.53^*c*^
Day 28 HR (95% CI)	0.61 (0.13–2.88)	1.0	
Day 55 deaths, *n* (%)	2 (14)	11 (27)	0.27^*c*^
Day 55 HR (95% CI)	0.44 (0.10–1.98)	1.0	
Respiratory support^*e*^			
LFNC days, median no. (IQR)	1 (0–3)	1 (0–3)	0.57^*f*^
Noninvasive ventilation days, median no. (IQR)	1 (0–3)	1 (0–8)	0.58^*f*^
Invasive ventilation days, median no. (IQR)	0 (0–0)	0 (0–6)	0.23^*f*^
Any respiratory support days, median no. (IQR)	4 (2–12)	6 (2–22)	0.45^*f*^
No. of patients with WHO clinical status shown on day 14/day 28^*g*^			0.07/0.40^*f*^
0–2	12/12	23/25	
3	0/0	0/3	
4	0/0	2/2	
5	1/0	5/0	
6	0/0	2/0	
7	1/0	7/1	
8	0/2	2/10	

a HR, hazard ratio; 95% CI, 95% confidence interval; IQR, interquartile range; LFNC, low-flow nasal cannula; NA, not available.

b HRs and 95% CIs for hospital discharge from first CCP infusion until days 14, 28, and 55 were estimated by the Fine-Gray method, accounting for competing risk of death.

c HRs and 95% CIs for time to death from first CCP infusion until days 14, 28, and 55 were based on the Cox proportional hazards model. *P* values were based on the log rank test.

d HR not estimable due to number of events.

e Cumulative number of days of respiratory support through day 55 post-first CCP infusion; any respiratory support includes LFNC and noninvasive and invasive ventilation.

f *P* values based on Wilcoxon rank sum test.

g The WHO clinical status scores shown are based on an 8-point ordinal scale.

### Safety outcomes.

By day 14 postinfusion, 13 (93%) participants receiving a high titer and 37 (90%) receiving the standard titer experienced at least one adverse event. Most adverse events were categorized as mild (grade 1) or moderate (grade 2) in severity regardless of titer received. Through 28 days, 27 participants (49%) experienced an adverse event of grade 3 or greater. Among these participants, 29% (4 of 14) receiving a high titer and 56% (23 of 41) receiving the standard titer had adverse events of grade 3 or greater (risk ratio, 0.51; 95% CI, 0.21 to 1.22; Fisher’s *P* = 0.12). Cumulative adverse events graded as greater than severe (i.e., life threatening and/or fatal) through 14 days occurred in 0 (0.0%) participants receiving a high titer and 12 (29%) receiving the standard titer (*P* = 0.02) (Table 3). There were no adverse events directly attributable to the CCP infusion in any of the study participants. The full list of adverse events through day 28 is presented in Table S1 in the supplemental material.

**TABLE 3:** Primary safety outcomes stratified by nAb titer of CCP received and days since first transfusion^*a*^

Adverse event	Result for nAb titer of CCP, no. (%)		Risk difference (95% CI)	Risk ratio (95% CI)
	High (*n* = 14)	Standard (*n* = 41)		
Through day 14^*b*^				
Any	13 (93)	37 (90)	0.03 (−0.14 to 0.19)	1.03 (0.86 to 1.23)
Mild (grade 1)	11 (79)	26 (63)	0.15 (−0.11 to 0.41)	1.24 (0.87 to 1.77)
Moderate (grade 2)	9 (64)	32 (78)	−0.14 (−0.42 to 0.14)	0.82 (0.54 to 1.26)
Severe (grade 3)	4 (29)	21 (51)	−0.23 (−0.51 to 0.06)	0.56 (0.23 to 1.35)
Life threatening	0 (0)	11 (27)	**−0.27 (−0.40 to −0.13)**	Undefined^*d*^
Fatal	0 (0)	3 (7)	−0.07 (−0.15 to 0.01)	Undefined^*d*^
Through day 28^*c*^				
Any	13 (93)	37 (90)	0.03 (−0.14 to 0.19)	1.03 (0.86 to 1.23)
Mild (grade 1)	11 (79)	27 (66)	0.13 (−0.13 to 0.39)	1.19 (0.84 to 1.70)
Moderate (grade 2)	9 (64)	32 (78)	−0.14 (−0.42 to 0.14)	0.82 (0.54 to 1.26)
Severe (grade 3)	4 (29)	23 (56)	−0.28 (−0.56 to 0.01)	0.51 (0.21 to 1.22)
Life threatening	0 (0)	12 (29)	**−0.29 (−0.43 to −0.15)**	Undefined^*d*^
Fatal	2 (14)	10 (24)	−0.10 (−0.33 to 0.12)	0.59 (0.15 to 2.36)

a 95% CI, 95% confidence interval. Results in boldface are statistically significant with a *P* value of <0.05. Data are shown as number (percentage) unless otherwise indicated.

b Seventy-nine percent (11/14) of high-titer and 63% (26/41) of standard-titer CCP patients had complete follow-up through day 14.

c Sixty-four percent (9/14) of high-titer and 44% (18/41) of standard-titer CCP patients had complete follow-up through day 28.

d Given no events in participants receiving high titer, the risk ratio is undefined.

10.1128/mbio.01751-22.3 TABLE S1 SAEs and AEs through day 28. ***, for non-SAEs, only AEs that occurred in at least 5% of either arm were reported. An AE had to occur in one high-titer participant or two standard-titer participants to be reported in this table. Download Table S1, DOCX file, 0.2 MB. Copyright © 2022 Bartelt et al. 2022 Bartelt et al. https://creativecommons.org/licenses/by/4.0/ This content is distributed under the terms of the Creative Commons Attribution 4.0 International license.

### Antibody content of CCP infused and in recipients prior to CCP infusion.

The median nAb titer of CCP units among participants receiving a high titer was 1:1,080 (IQR, 1:827 to 1:1,727; full range, 1:667 to 1:2,910) compared to 1:316 (IQR, 1:204 to 1:404; full range, 1:161 to 1:461) among participants receiving a standard titer (*P* < 0.0001) (Fig. S1). Additional assays were also performed on the CCP units. The median nAb titers of pseudovirus neutralization assays were higher among high-titer recipients (1:1,380; full range, 1:113 to 1:29,199) than standard-titer recipients (1:593; full range, 1:161 to 1:3,455) (*P* < 0.01), but there was some overlap between the two. Similarly, median anti-RBD IgG titers were 1:1,280 (full range, 1:320 to 1:4,800) in the high-titer recipients and 1:640 (full range, 1:40 to 1:2,520) in the standard-titer recipients (*P* < 0.01). In contrast, there was no difference in the index values for anti-N IgG between those receiving high and standard nAb: 6.36 (range, 3.92 to 6.82) and 5.57 (range, 0.72 to 8.52), respectively (*P* = 0.10). Spearman’s rank correlation coefficients for nAb and either pseudovirus, anti-RBD IgG, or anti-N IgG were 0.63 (95% CI, 0.42 to 0.77; *P* < 0.0001), 0.41 (95% CI, 0.15 to 0.61; *P* < 0.05), and 0.24 (95% CI, −0.03 to 0.48; *P* = 0.07), respectively.

10.1128/mbio.01751-22.1 FIG S1 Serological repertoire of CCP units in primary (as-treated), per-protocol and intention-to-treat group assignments. (A) As-treated 50% neutralizing antibody (NT_50_), RBD IgG, and nucleocapsid (N) IgG titer ranges, respectively, for high-titer (H; *n* = 14) and standard-titer (S; *n* = 41) CCP groups as indicated. (B) Per-protocol neutralizing antibody (NT_50_), RBD IgG, and nucleocapsid (N) IgG titer ranges, respectively, for high-titer (H; *n* = 14) and standard-titer (S; *n* = 28) CCP groups. (C) Intent-to-treat neutralizing antibody (NT_50_), RBD IgG, and nucleocapsid (N) IgG titer ranges, respectively, for high-titer (H; *n* = 27) CCP and standard-titer (S; *n* = 28) CCP groups. Medians are shown, analysis was done with the Mann-Whitney test, and *P* values for comparison between H and S groups are shown. Download FIG S1, DOCX file, 0.2 MB. Copyright © 2022 Bartelt et al. 2022 Bartelt et al. https://creativecommons.org/licenses/by/4.0/ This content is distributed under the terms of the Creative Commons Attribution 4.0 International license.

Among 34 patients with sera available prior to CCP infusion, 3 of 7 patients receiving a high titer and 12 of 27 receiving the standard titer had undetectable neutralizing antibody levels. Median neutralizing antibody titers were 1:26 (full range undetectable, 1:1,216) (*n* = 7) and 1:24 (full range undetectable, 1:743) (*n* = 27) in patients receiving high and standard titers, respectively (*P* = 0.98) (Table 1). In this subset (*n* = 34), 21% had a preinfusion nAb titer of >1:160, with 4, 3, 15, and 12 patients in the preinfusion/CCP nAb titer categories of >1:160/high, >1:160/standard, ≤1:160/high, and ≤1:160/standard, respectively (Table 4). Using the ≤1:160/standard as a reference, there was an interaction between the recipient preinfusion and CCP donor nAb titer such that the ≥1:160/high group had the greatest estimate of earlier time to discharge (hazard ratio, 4.15; 95% CI, 1.50 to 11.46), with the >1:160/standard and ≤1:160/high groups appearing intermediate. Similar trends were seen with mortality hazard ratio estimates, but with wide confidence intervals due to the low sample size.

**TABLE 4:** Exploratory clinical outcomes on day 55 post-CCP infusion among patients with available preinfusion anti-SARS-CoV-2 nAb titer^*a*^

CCP nAb titer group	Preinfusion nAb titer (*n* = 34)	*n*	Hospital discharge HR (95% CI)^*b*^	Deaths, no. (%)	Mortality HR (95% CI)^*c*^
High (>1:640)	>1:160	4	4.15 (1.50–11.46)	0 (0)	NA^*d*^
	Low (≤1:160)	15	1.64 (0.66–4.10)	3 (20)	0.27 (0.06–1.17)
Standard (≥1:160–1:640)	>1:160	3	1.20 (0.27–5.24)	1 (33)	0.56 (0.06–4.84)
	Low (≤1:160)	12	1.0	5 (42)	1.0

a HR, hazard ratio; 95% CI, 95% confidence interval; NA, not available.

b HRs and 95% CIs for hospital discharge from first CCP infusion until day 55 were estimated using one model with the standard-nAb-titer CCP and low (<1:160) preinfusion nAb titer as the referent, with the Fine-Gray method, accounting for competing risk of death.

c HRs and 95% CIs for time to death from first CCP infusion until day 55 were estimated using one model with low CCP and low preinfusion titer as the referent with a Cox proportional hazards model.

d Not estimable due to the available sample size.

### Secondary analyses.

In addition to our primary as-treated analyses, we also performed intention-to-treat (ITT) and per-protocol (PP) analyses. Using the ITT approach, analysis of the 13 participants who received standard titer based on their high-titer randomization assignment resulted in the nAb infused CCP titers overlapping between the two groups (Fig. S1) and a loss of separation in time to hospital discharge (Fig. S2). In the PP approach, which removed the 13 participants from analysis, the nAb infused CCP titers did not overlap (Fig. S1), and the high-titer assignment group had a shorter time to hospital discharge, but this was not statistically significant (Gray’s *P* = 0.3029) (Fig. S2).

10.1128/mbio.01751-22.2 FIG S2 Cumulative incidence curves in CCP recipients grouped by per-protocol and intention-to-treat group assignments. Time to hospital discharge from first CCP infusion until day 55 by CCP titer received per protocol (A to C) and intent to treat (D to F). Per-protocol (A) and intent-to-treat (D) nAbs with death as a competing event were estimated using the Aalen-Johansen estimator with Gray’s test with rho = 0. Shown are stacked cumulative incidence curves for death (dark gray), hospital discharge (light gray), and remaining hospitalized (blue) as competing risks among patients receiving high-titer CCP (B) and standard-titer CCP (C). Shown are stacked cumulative incidence curves for death (dark gray), hospital discharge (light gray), and remaining hospitalized (blue) as competing risks, treating all patients randomized to high-titer CCP that instead received standard-titer CCP as high-titer (E) and standard-titer CCP (F). Download FIG S2, DOCX file, 0.1 MB. Copyright © 2022 Bartelt et al. 2022 Bartelt et al. https://creativecommons.org/licenses/by/4.0/ This content is distributed under the terms of the Creative Commons Attribution 4.0 International license.

## DISCUSSION

To our knowledge, this is the first report of the effectiveness and safety of preassigned CCP defined as high (>1:640) and standard (1:160 to 1:640) nAb titers. We used these titer range cutoffs based on the initial FDA guidance that CCP be defined as having an nAb titer of ≥1:160, the internal variability of the nAb assay being ~2-fold, and our published findings that >1:640 represented the top quartile of CCP donors in our program (6). We found that patients receiving a high titer experienced accelerated time to recovery to hospital discharge and a trend toward lower mortality through 55 days postinfusion. Adverse events were common in both groups, but were reflective of underlying pathologies attributable to COVID-19 and not from the intervention. Despite a small sample size, unique strengths of our study include predefined titer assay assignments using a wild-type (WT) nanoluciferase (nLuc) SARS-CoV-2 viral neutralization assay that is a direct measure of functionally neutralizing antibody and has been used in the development of FDA-authorized mRNA vaccines (14) and implementation after corticosteroids and remdesivir had become standard institutional clinical practice and other therapies (like hydroxychloroquine and lopinavir/ritonavir) had fallen out of favor.

Observations that RBD-targeted antibodies have high viral neutralization potential paved the way for rapid development of highly effective vaccines and SARS-CoV-2-directed monoclonal antibody therapies (15). Similarly, we show much stronger correlation between nAb titers and RBD IgG-binding titers than N IgG index in this cohort. However, overlap exists between RBD IgG end titers and the nAb antibody titer patients received (high and standard) (see Fig. S1 in the supplemental material), supporting other findings that a single antibody-binding assay is not fully representative of global antiviral properties in CCP. Discrepancies in low-RBD but high-nAb-titer CCP may arise from individuals with unmeasured potent nAb targeted at epitopes outside the RBD, like the N-terminal domain (16). In contrast, high-RBD but lower-nAb-titer CCP could arise from RBD-directed antibodies that poorly compete with SARS-CoV-2 for ACE2 receptor binding. In either case, relying exclusively on a single target antibody ELISA index or end titer could lead to misclassification of CCP with high neutralizing function and therefore hinder interpretation of CCP clinical studies. These findings suggest the need to support infrastructure necessary to more rapidly deploy and scale antibody assays that directly measure antiviral function so convalescent plasma (CP) can be better characterized when used emergently in future pandemics.

Significant heterogeneity in clinical therapy studies, such as illness duration and severity, baseline participant characteristics, and temporal changes in standard of care, is not unexpected, especially amid a rapidly shifting pandemic like COVID-19. However, while most trials target a precisely defining therapeutic dose that is standardized in the intervention arm, the wide interindividual variability in CCP coupled with a lack of standardized assays for measuring antiviral and other functional properties precluded this process for CCP. Thus, several of the largest CCP trials used nAb titer-undefined plasma. For example, RECOVERY (*n* = 5,795 participants) did not report CCP nAb titer (17), RE-MAP CAP (ClinicalTrials registration no. NCT02735707) (*n* = 1,075 CC participants) measured nAb titer data on the majority (but not all) of CCP units (median, ~1:160 to 1:175), but the trial’s focus on critically ill patients at very late stages of COVID-19 (median 43 days after hospital admission) is an outlier beyond the window where any COVID-19-directed therapeutics are now being deployed (18), and CONCOR-1 enrolled 346 participants in the CCP group, but found significant heterogeneity in clinical outcomes driven by plasma suppliers that also varied in nAb titer ranges (4). Among 26 phase 2 or 3 CCP trials identified in PubMed between 2020 and 2022, 18 measured nAb on all units reported (*n* = 2,692 CCP participants), whereas the other 8 either did not perform an end titer assay (19), reported nAb titer in only a subset of units (18, 20), or did not report nAb titer (3, 21–24) (*n* = 7,079 CCP participants). Using in-study mortality as the most consistently reported primary or secondary outcome, aggregate risk ratios for death in CCP-treated-participants versus no-CCP comparator groups in trials reporting incomplete or undefined nAb titer (*n* = 8), median nAb at <1:160 (*n* = 7) (4, 25–30), or nAb at ≥1:160 (*n* = 11) (2, 4, 5, 31–38) were 0.99 (0.93 to 1.04), 1.04 (0.86 to 1.26), and 0.88 (0.73 to 1.05), respectively (Table S2). The median nAb titer in four studies exceeded 1:320 (39), but only one other study reported a median nAb titer of >1:640 in a CCP intervention arm (32–34, 39). Aggregate risk ratios for mortality increasingly favored higher-nAb-titer CCP compared with no CCP (or lower nAb titer in the CoronaVirus Inactivating Plasma [CoVIP] study [ClinicalTrials registration no. NCT04524507]), but with imprecise estimates due to small study size. While larger trials with CCP nAb at >1:640 are needed to establish if this very-high-titer CCP would have greater clinical benefit, this stratified comparison of CCP studies based on the nAb titer infused does raise an important need to take caution in interpretation of CCP study outcomes, even among the largest multicenter studies with precise statistical measurements but limited direct measurements of the functional properties of the therapy.

10.1128/mbio.01751-22.4 TABLE S2 Mortality (through latest time point measured) in randomized control trials of CCP in hospitalized adults organized by neutralizing antibody titer. Abbreviations: CCP, COVID-19 convalescent plasma; nAb, neutralizing antibody; NR, not reported; SOC, standard of care; FFP, fresh frozen plasma. ^^^A minimum nAb was required to qualify CCP for the study. ^*^nAb titers extrapolated from scatter plot. ^#^All are >1:160, and 80% are >1:320 median NR. Relative risks (95% CI) were independently calculated from published values using R Studio. For Avendaño-Sola et al. (5), the Haldane-Anscombe correction was used to account for zero mortalities in the treatment group. Download Table S2, DOCX file, 0.2 MB. Copyright © 2022 Bartelt et al. 2022 Bartelt et al. https://creativecommons.org/licenses/by/4.0/ This content is distributed under the terms of the Creative Commons Attribution 4.0 International license.

Our findings provide important proof of concept that potent neutralizing antibody-based therapies may have a role in the management of patients who rapidly progress to severe COVID-19 and/or miss outpatient therapy windows prior to hospitalization. A *post hoc* analysis in RECOVERY showed a benefit of monoclonal antibody therapy among patients hospitalized with COVID-19 who had not yet developed a seroresponse (17). Similarly, there was evidence of trends of benefit among certain subgroups of patients in large CCP RCTs, including immunosuppressed patients and those treated earlier in the course of disease (40). In our exploratory analysis, we found that earlier patient seroconversion was associated with better outcome, and the most advantageous outcomes were among those who also received the highest-antibody-content CCP. These findings may indicate synergy between the nAb in CCP and the recipient preinfusion nAb. Indeed, in an open-label CCP study, an endogenous recipient preinfusion neutralizing antibody titer of >1:100 was associated with lower plasma viral loads and a donor neutralizing antibody titer of >1:250 was associated with more rapid decay in nasopharyngeal viral loads (41). Further investigation is needed to better understand the potential for polyclonal antibodies, including those with nonneutralizing as well as neutralizing functions, to work additively or synergistically together and whether polyclonal antibodies might decrease the risk of treatment-emergent mutations during RBD-directed monoclonal antibody therapy that can rapidly lead to antibody-resistant variants and subsequently prolonged viral shedding (42). In addition, a more complex mixture of neutralizing antibodies, such as CCP from vaccinated individuals after experiencing Omicron breakthrough infection, might retain activity against emerging variants of concern, like Omicron subvariants that were not inhibited by several available commercial monoclonal antibodies at the time of their emergence (43). Broader characterization of these antibody responses could inform potential prevention and treatment applications for patients with B cell depletion who are incapable of generating an endogenous antibody response.

Our study has several limitations. First, like several prior CCP RCTs, we had difficulty reaching target enrollment for each of our titer-defined groups. Our inability to identify enough donors with high-titer CCP resulted in 50% of the patients randomized to the high-nAb-titer group instead receiving standard-nAb-titer CCP. Given we were unable to follow the original randomization assignment, we chose to analyze the primary study results using a quasiexperimental approach as an as-treated analysis (44), including adjusting for possible confounding. Specifically, we contrasted baseline patient characteristics by CCP titer received and did not detect any statistically significant imbalances (Table 1). We also adjusted the primary results for a number of factors, including respiratory support, sex, age, race/ethnicity, and comorbidity at baseline (Fig. 3), one at a time in multivariate models. Adjustment did not appreciably affect the estimate or its precision. Since treatment assignment deviation was based solely on CCP availability and investigators and treating providers remained blind to the treatment received, we did not observe any confounding, and our adjusted analyses were consistent with our crude results. Our confidence in our findings is supported by the results of the PP and ITT analyses that showed similar trends (but with greater imprecision given the more limited sample size) in the former and no apparent effect in the latter, which represented an admixture of nearly 50% in those randomized to receive a high titer actually receiving CCP within the standard-titer range. Second, we could not adequately power a three-arm study and we were unable to include a CCP-free group. Third, we are underpowered in this single-center study to reach a statistical conclusion for outcomes like inpatient mortality. Fourth, since D614G was the most common variant in circulation during our study and CCP collection, we cannot extrapolate our findings to other variants, like Omicron subvariants. Finally, this study evaluates the neutralizing antibody responses to COVID-19 infection, but this is only part of the global immune response, which also includes T cell responses and B cell responses.

As SARS-CoV-2 variants continue to emerge and spread, it is possible that CCP will remain an alternative therapeutic should others be unavailable or ineffective against an emergent variant. We suggest that a definition of high-titer CCP exceeding >1:640 gives the greatest confidence in benefit potential and that future CCP trials deliberately use direct measures of the functional anti-SARS-CoV-2 properties to more precisely preassign CCP and avoid infusing low-titer CCP that is unlikely to have clinical benefit.

## MATERIALS AND METHODS

### Study design and participants.

The CoronaVirus Inactivating Plasma (CoVIP) study was originally designed as a double-blind, randomized phase 2 trial of COVID-19 convalescent plasma contrasting treatment with a defined neutralizing antibody titer at least 4-fold higher than the recommended minimum (1:160), defined as high titer (>1:640) versus standard titer (1:160 to 1:640) (45). CoVIP was conducted at The University of North Carolina at Chapel Hill (UNC) in accordance with FDA IND 22282 (ClinicalTrials.gov registration no. NCT04524507). The original protocol was designed to enroll 56 participants in a 1:1 randomization schema to receive either high- or standard-titer CCP.

Eligible participants were adults ≥18 years of age hospitalized for SARS-CoV-2 infection defined as laboratory confirmation with a specific SARS-CoV-2 PCR and clinical symptoms of COVID-19. COVID-19 symptom onset had to be <8 days prior to admission and defined as having one or more respiratory or gastrointestinal symptoms, including but not limited to cough, shortness of breath, difficulty breathing, sore throat, loss of taste, loss of smell, diarrhea, nausea, or vomiting. Patients without subjective or objective fever (≥38.0°C) but with other symptoms consistent with COVID-19 were enrolled or excluded by study primary investigator discretion. Key exclusion criteria were ongoing or prior receipt of immune-based therapies, including pooled immunoglobulin within the past 30 days, antibody or T-cell-based therapies specific to SARS-CoV-2, contraindication to blood transfusion, absence of ABO-compatible plasma, and inability to infuse the first unit of CCP within 48 h of enrollment.

Participants or legally authorized representatives (LARs) provided written (electronic or paper) informed consent. Separate consent for blood transfusion was also obtained according to institutional standard operating procedures. The clinical trial protocol and informed consent (in both English and Spanish) were approved by the Institutional Review Board at UNC-CH (IRB 20-1544) (ClinicalTrials.gov registration no. NCT04524507). The study was done in accordance with the principles stated in the Declaration of Helsinki and Good Clinical Practice guidelines.

### Intervention.

Each participant was to receive 2 U of CCP (a total of ~400 to 500 mL) within 48 h of randomization. The 2 U could be infused up to 24 h apart. An optional third unit of CCP from the same donor was allowed if available, but no participant received >2 U of CCP. All eligible participants also received institution-guided standard of care.

### Randomization and masking.

Participants were randomly assigned in a 1:1 ratio to receive either high- or standard-titer CCP, with randomization stratified by ABO blood group, using permuted block group randomization. During the conduct of the study, 13 participants randomized to receive high-titer CCP received standard-titer CCP because appropriate ABO-compatible high-titer CCP was not available. The decision to allow patients access to this therapy was made by the primary investigators, and a protocol deviation was approved by the IRB at a time when treatment options were limited and there was equipoise regarding CCP titer. Unmasking was limited to the Blood Bank physician coinvestigator, who needed access to titer levels to provide appropriate ABO-matched CCP to participants. All study coordinators, clinical physician investigators, treating physicians, participants, and other members of the participants’ health care teams remained masked to treatment assignment. All units of CCP contained a standardized label without designation of neutralizing antibody titer such that health care providers, study personnel, and participants remained masked to titer assignment.

We used an honest-broker screening procedure centralized in the Division of Infectious Diseases at UNC-CH for referrals. In this procedure, a non-study staff screener filtered new admissions for COVID-19 twice daily and arbitrated eligibility to this and other ongoing intervention studies in the hospital. Study staff were contacted with potential eligible participants. Study staff also interfaced with treating teams to confirm appropriateness to approach for the study.

### Procedures.

**(i) Donors.** Convalescent plasma from volunteers who had recovered from SARS-CoV-2 infection was collected by apharesis in the UNC Blood Donation Center (BDC) in accordance with the UNC BDC standard operating procedures and stored on-site. All donations adhered to FDA guidance for CCP, and further details for CCP donor recruitment have been previously described (6). Documentation of SARS-CoV-2 infection by nucleic acid amplification test or antibody test was required to qualify for donation. For measurement of anti-SARS-CoV-2 antibodies, blood from the apheresis diversion pouch was collected and transported on ice for fractionation into serum and cellular components as approved by UNC IRB 20-1141. Mean time from diagnosis to CCP collection for this group of donors was 84 days (range, 19 to 176).

**(ii) Anti-SARS-CoV-2 antibody assays.** Neutralizing antibody titers were measured using a SARS-CoV-2-WA1 viral reporter neutralization assay expressing a nanoluciferase (nLuc) gene and were recovered using reverse genetics as previously described (46). Neutralization assays were performed as previously described (6, 12, 46). The same assay has been used to assess mRNA vaccine effectiveness (14). The high-nAb-titer minimum was set to be at least 4-fold higher than the initial FDA recommendation of ≥1:160 and to exceed the upper limit of viral neutralization plaque assay-based titers reported at the time of study design (high titer, >1:640) (47). The minimum threshold for CCP in the standard-titer group was set at the initial FDA recommendation (≥1:160) and the maximum to align with the high-titer cutoff (standard titer, 1:160 to 1:640).

Binding-antibody assays to SARS-CoV-2 spike protein receptor binding domain (RBD) and nucleocapsid (N) IgG (Abbott Laboratories) were done as previously described (6). Briefly, RBD IgG, IgA, and IgM endpoint titers were determined on heat-inactivated serum samples in an in-house-developed enzyme-linked immunosorbent assay format starting with a titer of 1:20 (12). Nucleocapsid IgG levels were measured in the EUA-approved Abbott SARS-CoV-2 IgG assay in a Clinical Laboratory Improvement Amendments (CLIA)-certified laboratory using the Abbott Architect i2000SR immunoassay analyzer, which is further described in reference 6.

**(iii) Recipients.** Participants were monitored by hospital staff during and after each CCP infusion according to standard hospital operating procedures. Clinical symptoms (vital signs and physical exam as documented by the treating teams), 8-point WHO ordinal status, laboratory data, and adverse event assessments were obtained on day 1, day 3 (±1), day 7 (±2), day 14 (±2), day 21 (±2), and day 28 (±7). Clinical symptoms were also obtained for participants who elected into an extended study protocol with visits on days 49 (±10), 90 (±14), and 180 (±14). Serum and/or plasma was available from 34 participants (27 in the standard-titer group and 7 in the high-titer group) prior to CCP administration. To minimize exposures and use of protective equipment by staff and to minimize sample collection burdens on participants, whenever possible, blood and mucosal lining fluid samples were obtained from remnants of samples collected for clinical purposes. CCP recipients were infected with SARS-CoV-2 during the wild-type D614G phase.

### Outcomes.

The primary clinical effectiveness endpoint was the days to hospital discharge following the first dose of CCP. Exploratory clinical endpoints included mortality, changes in clinical severity scores (e.g., WHO ordinal clinical status scale), and days of supplemental oxygen, noninvasive ventilation/high-flow oxygen, and/or invasive ventilation/extracorporeal membrane oxygenation (ECMO) requirement. The primary safety endpoint was the cumulative incidence of serious adverse events (SAEs) at study days 14 and 28 after the first CCP infusion. All participants were followed until hospital discharge (day 55 post-first infusion), with an additional 6 months of follow-up obtained through review of the institutional electronic health record.

### Statistical analysis.

Given that 50% of the participants randomized to the high-titer group instead received standard-titer CCP, we performed unadjusted and adjusted as-treated analyses focusing on the biological differences in the interventions provided—in effect treating the study as a quasiexperimental study, accounting for confounding in adjusted analyses (44). We used descriptive statistics to describe patient demographic and clinical characteristics at baseline (date of randomization/first unit of CCP). We compared distributions of baseline characteristics by CCP titer received using Fisher’s exact, Pearson’s χ^2^, and Kruskal-Wallis tests, as appropriate. We contrasted primary safety endpoints through day 14 and day 28 by CCP titer received using risk ratios and risk differences and 95% confidence intervals as measures of precision. To compare nAb and binding Ab titers by CCP titer received, we used the Mann-Whitney U test.

For the primary clinical endpoint, time from first CCP infusion until hospital discharge, we contrasted the cumulative incidence functions by CCP titer received, accounting for the competing risk of death using Aalen-Johansen estimator and Gray’s test with rho = 0. Hazard ratios and 95% confidence intervals for time to hospital discharge by CCP titer were estimated using the Fine-Gray method, accounting for the competing risk of death. Time to death by CCP titer received was compared using the log rank test, and hazard ratios were estimated using Cox proportional hazards models. The proportional hazards assumption was assessed for Cox regression models.

Given our primary analytic approach was an as-treated analysis, we evaluated for possible confounding of the hazard ratio for time to hospital discharge by CCP titer received for variables reported to increase risk of poor outcome in patients hospitalized with COVID-19: age, sex, race/ethnicity, respiratory support needs at enrollment, and presence of any comorbidity (hypertension, diabetes, obesity, cardiovascular disease, chronic pulmonary disease, solid tumor, hematologic malignancies, solid organ transplant, and hematologic stem cell transplantation). We first assessed whether there was an association between any of the possible confounders and receiving either high or standard CCP using standard descriptive statistics. We then assessed whether any possible confounder was associated with our primary clinical endpoint by estimating unadjusted hazard ratios. We also fit separate multivariable models for time to hospital discharge by CCP titer received for each potential confounder: for example, the hazard ratio adjusted for age is based on a model that includes only age as a confounder. We did not fit one full multivariable model including all potential confounders given the limited sample size.

In addition to our primary as-treated analyses, where participants were analyzed based on the titer they received, we also performed intention-to-treat (ITT) and per-protocol (PP) analyses of the primary clinical outcome of time to hospital discharge. For the ITT analyses, participants were analyzed based on their randomization assignment (*n* = 27 high titer and *n* = 28 standard titer). For the PP analyses, we only included in the analyses the participants who received the titer to which they were randomized (*n* = 14 high titer and *n* = 28 standard titer), removing from analyses the 13 participants randomized to the high-titer arm who received the standard titer.

All hypothesis testing was two sided. Statistical analyses were performed using SAS software, version 9.4 (SAS Institute) and R software, version 4.0.

### Data availability.

Deidentified individual participant-level clinical data and antibody assay data will be made available upon request immediately after publication and upon approval of the principal investigators. The study protocol, statistical analysis plan, the clinical study report, and summary outcomes are posted at ClinicalTrials.gov under no. NCT04524507. Analytic code is available upon request and approval of S.N.
