# Supplementary material for: Outcomes of Convalescent Plasma with Defined High versus Lower Neutralizing Antibody Titers against SARS-CoV-2 among Hospitalized Patients: CoronaVirus Inactivating Plasma (CoVIP) Study
Source: mBio. 2022 Sep 22;13(5):e01751-22. doi: 10.1128/mbio.01751-22 (PMC9601237; doi:10.1128/mbio.01751-22)
Supplement: TABLE S1 [file mbio.01751-22-s0001.docx]

**Supplementary Table 1: SAEs and AEs through day 28**
