# Supplementary material for: Outcomes of Convalescent Plasma with Defined High versus Lower Neutralizing Antibody Titers against SARS-CoV-2 among Hospitalized Patients: CoronaVirus Inactivating Plasma (CoVIP) Study
Source: mBio. 2022 Sep 22;13(5):e01751-22. doi: 10.1128/mbio.01751-22 (PMC9601237; doi:10.1128/mbio.01751-22)
Supplement: FIG S1 [file mbio.01751-22-s0003.docx]

**Figure S1. Serological repertoire of CCP units in primary (as-treated), per protocol and intention-to-treat group assignments.** A) As-treated neutralizing antibody (NT50), RBD IgG, and nucleocapsid (N) IgG titer ranges, respectively, for high titer (H, n=14) CCP, and standard titer (S, n=41) CCP groups as indicated. B) Per-protocol neutralizing antibody (NT50), RBD IgG, and nucleocapsid (N) IgG titer ranges, respectively, for high titer (H, n=14) CCP and standard titer (S, n=28) CCP groups. C) Intent-to-treat neutralizing antibody (NT50), RBD IgG, and nucleocapsid (N) IgG titer ranges, respectively, for high titer (H, n=27) CCP and standard titer (S, n=28) CCP groups. Medians shown, analysis done with Mann-Whitney test, p values between H and S groups shown.
