# Supplementary material for: Outcomes of Convalescent Plasma with Defined High versus Lower Neutralizing Antibody Titers against SARS-CoV-2 among Hospitalized Patients: CoronaVirus Inactivating Plasma (CoVIP) Study
Source: mBio. 2022 Sep 22;13(5):e01751-22. doi: 10.1128/mbio.01751-22 (PMC9601237; doi:10.1128/mbio.01751-22)
Supplement: FIG S2 [file mbio.01751-22-s0002.docx]

| **A.** | **B.** | **C.** |
| --- | --- | --- |
| 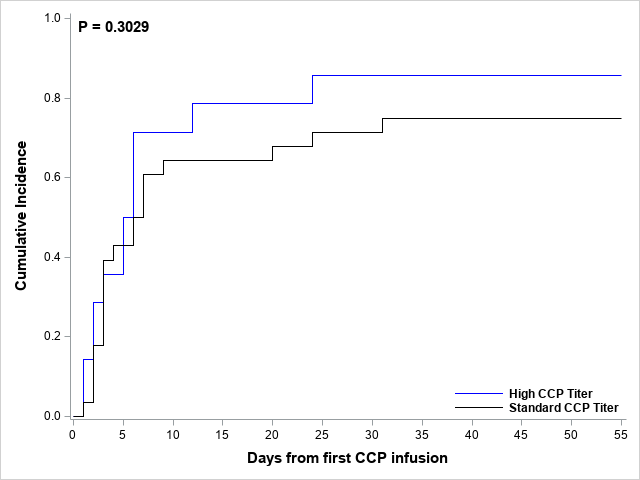 | 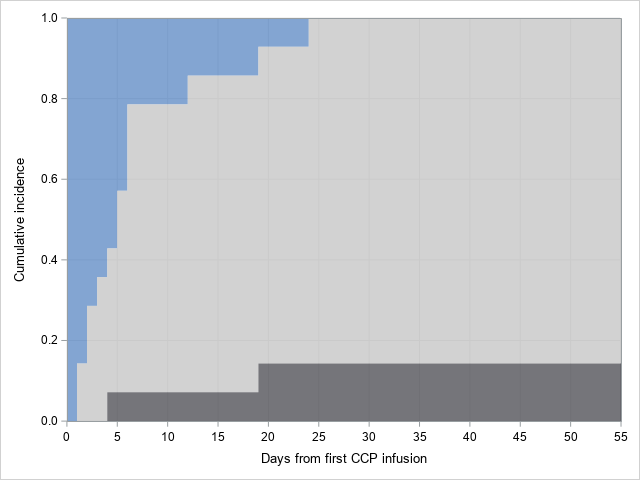 | 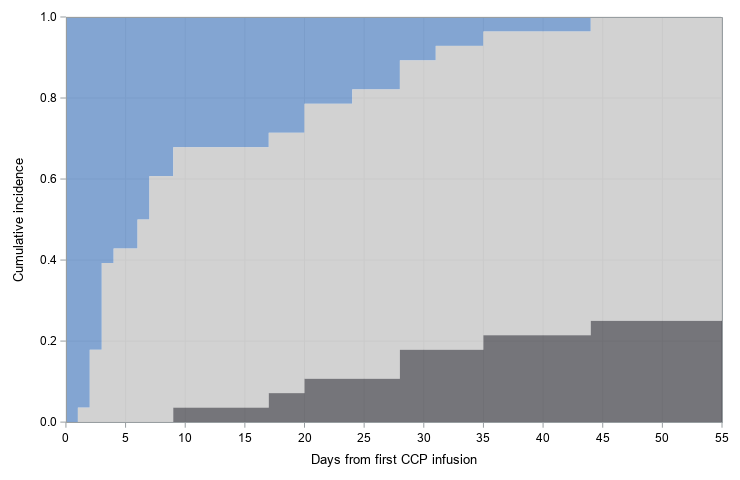 |
| **D.** | **E.** | **F.** |
| 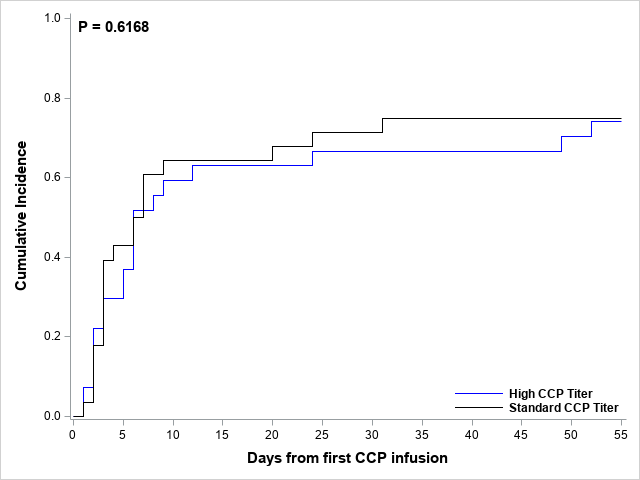 | 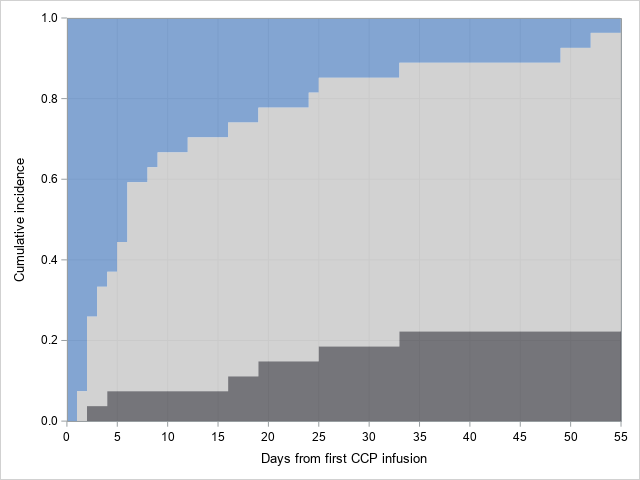 | 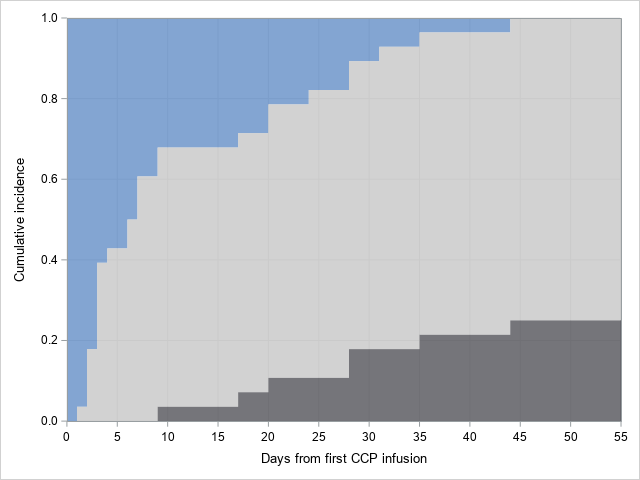 |

**Figure S2. Cumulative incidence curves in CCP recipients grouped by per protocol and intention-to-treat group assignments.**

Time to hospital discharge from first CCP infusion until day 55, by CCP titer received Per-Protocol (Panels A-C) and Intent-to-Treat (Panels D-F). Per-Protocol (Panel A) and Intent-to Treat (Panel D) with death as a competing event, estimated using the Aalen-Johansen estimator, with Gray’s test with rho=0. Stacked cumulative incidence curves for death (dark gray), hospital discharge (light gray) and remaining hospitalized (blue), as competing risks, among patients receiving high titer CCP (Panel B), and standard titer CCP (Panel C). Stacked cumulative incidence curves for death (dark gray), hospital discharge (light gray) and remaining hospitalized (blue), as competing risks, treating all patients randomized to high CCP titer that instead received standard titer CCP as high (Panel E), and standard titer CCP (Panel F).
