# Supplementary material for: Outcomes of Convalescent Plasma with Defined High versus Lower Neutralizing Antibody Titers against SARS-CoV-2 among Hospitalized Patients: CoronaVirus Inactivating Plasma (CoVIP) Study
Source: mBio. 2022 Sep 22;13(5):e01751-22. doi: 10.1128/mbio.01751-22 (PMC9601237; doi:10.1128/mbio.01751-22)
Supplement: TABLE S2 [file mbio.01751-22-s0004.docx]

**Supplementary Table 2: Mortality (through latest timepoint measured) in randomized control trials of CCP in hospitalized adults organized by neutralizing antibody titer**

| ***nAb titer defined in all units*** | Days from symptoms onset | Intervention  median nAb titer (IQR) | Comparison | Mortality Treatment | Mortality  Comparison | RR (95% CI) | Ref |  |
| --- | --- | --- | --- | --- | --- | --- | --- | --- |
| **median** **> 1:640 (n=2)** |  |  |  |  |  |  |  | |
| *CoVIP (this study)^* | 6 (4-8) | 1:1080 (1:667-1:2910) | 1:316 (1:161-1:461) | 2/14 (14%) | 11/41 (27%) | 0.53 (0.13-2.11) |  | |
| *Gharbharan et al (2020)^* | 10 (6-15) | 1:640 (1:320-1:1280) | SOC | 6/43 (14%) | 11/43 (26%) | 0.55 (0.22-1.34) | ^1^ | |
| **Aggregate** |  |  |  | **8/57 (14%)** | **22/84 (26%)** | **0.53 (0.26-1.11)** |  | |
| **> 1:320 (n=4)** |  |  |  |  |  |  |  | |
| *CoVIP (this study)^* | 6 (4-8) | 1:1080 (1:667-1:2910) | 1:316 (1:161-1:461) | 2/14 (14%) | 11/41 (27%) | 0.53 (0.13-2.11) |  | |
| *Gharbharan et al (2020)^* | 10 (6-15) | 1:640 (1:320-1:1280) | SOC | 6/43 (14%) | 11/43 (26%) | 0.55 (0.22-1.34) | ^1^ | |
| *Simonovich et al (2021)* | 8 (5-10) | 1:400 (1:90-1:800) | SOC | 25/228 (11%) | 12/105 (11%) | 0.96 (0.50-1.83) | ^2^ | |
| *Bennett-Guerrero et al (2021)* | 9 (6-18) | 1:334 (1:192-1:714) | FFP | 16/59 (27%) | 5/15 (33%) | 0.81 (0.36-1.86) | ^3^ | |
| **Aggregate** |  |  |  | **49/344 (14%)** | **39/204 (19%)** | **0.75 (0.51-1.10)** |  | |
| **median ≥ 1:160 (n=11)** |  |  |  |  |  |  |  | |
| *Gharbharan et al (2020)^* | 10 (6-15) | 1:640 (1:320-1:1280) | SOC | 6/43 (14%) | 11/43 (26%) | 0.55 (0.22-1.34) | ^1^ | |
| *Simonovich et al (2021)* | 8 (5-10) | 1:400 (1:90-1:800) | SOC | 25/228 (11%) | 12/105 (11%) | 0.96 (0.50-1.83) | ^2^ | |
| *Bennett-Guerrero et al (2021)** | 9 (6-18) | 1:334 (1:192-1:714) | FFP | 16/59 (27%) | 5/15 (33%) | 0.81 (0.36-1.86) | ^3^ | |
| *Sekine et al (2022)* | 10 (8-12) | 1:320 (1:160-1:960) | SOC | 18/80 (23%) | 13/80 (16%) | 1.38 (0.73-2.63) | ^4^ | |
| *Avendaño-Solà et al (2020)* | 8 (6-9) | 1:292 (1:238-1:451) | SOC | 0/38 (0%) | 4/43 (9%) | 0.14 (0.007-2.55) | ^5^ | |
| *van den Berg (2022)* | 9 (6-11) | 1:234 (1:194-1:304) | NS | 11/52 (21%) | 13/51 (25%) | 0.83 (0.41-1.68) | ^6^ | |
| *Menichetti et al* | 7.7 (5.0-9.0) | 1:226 (1:160-1:320) | SOC | 14/231 (6%) | 19/240 (8%) | 0.77 (0.39-1.49) | ^7^ | |
| *Devos et al (2022)^* | 7 (4-9) | >1:160^#^ | SOC | 28/314 (9%) | 14/163 (9%) | 1.04 (0.56-1.92) | ^8^ | |
| *O'Donnell et al (2021)* | 9 (7-11) | 1:160 (1:80-1:320) | FFP | 19/150 (13%) | 18/73 (25%) | 0.51 (0.29-0.92) | ^9^ | |
| *Körper et al (2021)* | 7 (2-9) | 1:160 (1:80-1:320) | SOC | 11/53 (21%) | 17/52 (33%) | 0.63 (0.33-1.22) | ^10^ | |
| *Begin et al. (2021) supplier 1* | 8 (5-10) | 1:160 (1:160-1:640) | SOC | 75/343 (22%) | 40/173 (23%) | 0.95 (0.67-1.33) | ^11^ | |
| **Aggregate** |  |  |  | **223/1591 (14%)** | **166/1038 (16%)** | **0.88 (0.73-1.05)** |  | |
| **Aggregate (1:160-1:320)** |  |  |  | **176/1261 (14%)** | **138/875 (16%)** | **0.89 (0.72-1.09)** |  | |
| **median <1:160 (n=7)** |  |  |  |  |  |  |  | |
| *Kirenga et al (2021)* | 7 (4-8) | 1:139.5 (84.3-195.4) | SOC | 10/69 (14%) | 8/67 (12%) | 1.21 (0.51-2.89) | ^12^ | |
| *De Santis et al (2022)* | 8 (7-10 | 1:128 (NR) | SOC | 11/33 (33%) | 25/71 (35%) | 0.95 (0.53-1.68) | ^13^ | |
| *Holm et al (2021)* | 7 (5-9) | 1:116 (NR) | SOC | 2/17 (12%) | 3/14 (21%) | 0.55 (0.11-2.84) | ^14^ | |
| *Ortigoza et al (2021)* | 7 (4-9) | 1:93 (1:48-1:213) | NS | 59/462 (13%) | 71/462 (15%) | 0.83 (0.60-1.15) | ^15^ | |
| *Bajpai et al (2020)* | < 4 | ≥1:80 (1:10-≥1:80) | FFP | 3/14 (21%) | 1/15 (7%) | 3.21 (0.38-27.3) | ^16^ | |
| *Begin et al (2021) suppliers 2,3,4* | 8 (5-10) | 1:80 (1:20-1:160) | SOC | 68/271 (25%) | 23/134 (17%) | 1.46 (0.96-2.24) | ^11^ | |
| *Agarwal et al (2020)* | 4 (3-7) | 1:40 (1:30 - 1:80) | SOC | 34/235 (14%) | 31/229 (14%) | 1.07 (0.68-1.68) | ^17^ | |
| **Aggregate** |  |  |  | **187/1101 (17%)** | **162/992 (16%)** | **1.04 (0.86-1.26)** |  | |
| ***incomplete or undefined nAb titer*** | | |  |  |  |  |  | |
| **CCP≤ day 10 from onset (n=3)** |  |  |  |  |  |  |  | |
| *Balcells et al (2020)* | 6 (4-7) | Incomplete | SOC | 5/28 (18%) | 2/30 (7%) | 2.68 (0.57-12.7) | ^18^ | |
| *Bar et al (2021)* | 6 (4-9) | Not reported | SOC | 2/40 (5%) | 10/39 (26%) | 0.20 (0.05-0.83) | ^19^ | |
| *RECOVERY* | 9 (6-12) | Not reported | SOC | 1399/5795 (24%) | 1408/5763 (24%) | 0.99 (0.93-1.05) | ^20^ | |
| ***Aggregate (≤10d)*** |  |  |  | ***1406/5863 (24%)*** | ***1420/5832 (24%)*** | ***0.98 (0.92-.1.05)*** |  | |
| **CCP> day 10 from onset or NR (n=5)** |  |  |  |  |  |  |  | |
| *RE-MAP CAP (2021)* | 43 (24-79) | Incomplete | SOC | 401/1075 (37%) | 347/904 (38%) | 0.97 (0.87-1.09) | ^21^ | |
| *Ray et al (2020)* | Not reported | Not quantified | SOC | 10/40 (25%) | 14/40 (35%) | 0.71 (0.36-1.41) | ^22^ | |
| *Li et al (2020)* | 30 (19-38) | Not reported | SOC | 8/51 (16%) | 12/50 (24%) | 0.65 (0.29-1.46) | ^23^ | |
| *AlQahtani et al (2020)* | Not reported | Not reported | SOC | 1/20 (5%) | 2/20 (10%) | 0.50 (0.05-5.08) | ^24^ | |
| *Pouladzadeh et al (2021)* | Not reported | Not reported | SOC | 3/30 (10%) | 5/30 (17%) | 0.60 (0.16-2.29) | ^25^ | |
| ***Aggregate (>10d)*** |  |  |  | ***423/1216 (35%)*** | ***380/1044 (36%)*** | ***0.96 (0.86-1.07)*** |  | |
| **Aggregate (any nAb titer undefined)** |  |  |  | **1829/7079 (26%)** | **1800/6876 (26%)** | **0.99 (0.93-1.04)** |  | |

NOTE: Abbreviations: CCP=Covid-19 Convalescent Plasma, nAb=neutralizing antibody, NR=not reported, SOC=standard of care, FFP= fresh frozen plasma. ^^^a minimum nAb was required to qualify CCP for the study. ^*^nAb titers extrapolated from scatter plot. # all >1:160, 80% >1:320 median NR. Relative risks (95% CI) were independently calculated from published values using R studio. For *Avendaño-Solà et al (2020)* the Haldane-Anscombe correction was used to account for zero mortalities in the treatment group.

**References**

1 Gharbharan, A. *et al.* Effects of Treatment of Coronavirus Disease 2019 With Convalescent Plasma in 25 B-Cell-Depleted Patients. *Clin Infect Dis* **74**, 1271-1274, doi:10.1093/cid/ciab647 (2022).

2 Simonovich, V. A. *et al.* A Randomized Trial of Convalescent Plasma in Covid-19 Severe Pneumonia. *N Engl J Med* **384**, 619-629, doi:10.1056/NEJMoa2031304 (2021).

3 Bennett-Guerrero, E. *et al.* Severe Acute Respiratory Syndrome Coronavirus 2 Convalescent Plasma Versus Standard Plasma in Coronavirus Disease 2019 Infected Hospitalized Patients in New York: A Double-Blind Randomized Trial. *Crit Care Med* **49**, 1015-1025, doi:10.1097/CCM.0000000000005066 (2021).

4 Sekine, L. *et al.* Convalescent plasma for COVID-19 in hospitalised patients: an open-label, randomised clinical trial. *Eur Respir J* **59**, doi:10.1183/13993003.01471-2021 (2022).

5 Avendano-Sola, C. *et al.* A multicenter randomized open-label clinical trial for convalescent plasma in patients hospitalized with COVID-19 pneumonia. *J Clin Invest* **131**, doi:10.1172/JCI152740 (2021).

6 van den Berg, K. *et al.* Convalescent plasma in the treatment of moderate to severe COVID-19 pneumonia: a randomized controlled trial (PROTECT-Patient Trial). *Sci Rep* **12**, 2552, doi:10.1038/s41598-022-06221-8 (2022).

7 Menichetti, F. *et al.* Effect of High-Titer Convalescent Plasma on Progression to Severe Respiratory Failure or Death in Hospitalized Patients With COVID-19 Pneumonia: A Randomized Clinical Trial. *JAMA Netw Open* **4**, e2136246, doi:10.1001/jamanetworkopen.2021.36246 (2021).

8 Devos, T. *et al.* A randomized, multicentre, open-label phase II proof-of-concept trial investigating the clinical efficacy and safety of the addition of convalescent plasma to the standard of care in patients hospitalized with COVID-19: the Donated Antibodies Working against nCoV (DAWn-Plasma) trial. *Trials* **21**, 981, doi:10.1186/s13063-020-04876-0 (2020).

9 O'Donnell, M. R. *et al.* A randomized double-blind controlled trial of convalescent plasma in adults with severe COVID-19. *J Clin Invest* **131**, doi:10.1172/JCI150646 (2021).

10 Korper, S. *et al.* Results of the CAPSID randomized trial for high-dose convalescent plasma in patients with severe COVID-19. *J Clin Invest* **131**, doi:10.1172/JCI152264 (2021).

11 Begin, P. *et al.* Convalescent plasma for hospitalized patients with COVID-19: an open-label, randomized controlled trial. *Nat Med* **27**, 2012-2024, doi:10.1038/s41591-021-01488-2 (2021).

12 Kirenga, B. *et al.* Efficacy of convalescent plasma for treatment of COVID-19 in Uganda. *BMJ Open Respir Res* **8**, doi:10.1136/bmjresp-2021-001017 (2021).

13 De Santis, G. C. *et al.* High-Dose Convalescent Plasma for Treatment of Severe COVID-19. *Emerg Infect Dis* **28**, 548-555, doi:10.3201/eid2803.212299 (2022).

14 Holm, K. *et al.* Convalescence plasma treatment of COVID-19: results from a prematurely terminated randomized controlled open-label study in Southern Sweden. *BMC Res Notes* **14**, 440, doi:10.1186/s13104-021-05847-7 (2021).

15 Ortigoza, M. B. *et al.* Efficacy and Safety of COVID-19 Convalescent Plasma in Hospitalized Patients: A Randomized Clinical Trial. *JAMA Intern Med* **182**, 115-126, doi:10.1001/jamainternmed.2021.6850 (2022).

16 Bajpai, M. *et al.* Efficacy of convalescent plasma therapy in the patient with COVID-19: a randomised control trial (COPLA-II trial). *BMJ Open* **12**, e055189, doi:10.1136/bmjopen-2021-055189 (2022).

17 Agarwal, A. *et al.* Convalescent plasma in the management of moderate covid-19 in adults in India: open label phase II multicentre randomised controlled trial (PLACID Trial). *BMJ* **371**, m3939, doi:10.1136/bmj.m3939 (2020).

18 Balcells, M. E. *et al.* Early versus deferred anti-SARS-CoV-2 convalescent plasma in patients admitted for COVID-19: A randomized phase II clinical trial. *PLoS Med* **18**, e1003415, doi:10.1371/journal.pmed.1003415 (2021).

19 Bar, K. J. *et al.* A randomized controlled study of convalescent plasma for individuals hospitalized with COVID-19 pneumonia. *J Clin Invest* **131**, doi:10.1172/JCI155114 (2021).

20 Group, R. C. Convalescent plasma in patients admitted to hospital with COVID-19 (RECOVERY): a randomised controlled, open-label, platform trial. *Lancet* **397**, 2049-2059, doi:10.1016/S0140-6736(21)00897-7 (2021).

21 Writing Committee for the, R.-C. A. P. I. *et al.* Effect of Convalescent Plasma on Organ Support-Free Days in Critically Ill Patients With COVID-19: A Randomized Clinical Trial. *JAMA* **326**, 1690-1702, doi:10.1001/jama.2021.18178 (2021).

22 Ray, Y. *et al.* A phase 2 single center open label randomised control trial for convalescent plasma therapy in patients with severe COVID-19. *Nat Commun* **13**, 383, doi:10.1038/s41467-022-28064-7 (2022).

23 Li, L. *et al.* Effect of Convalescent Plasma Therapy on Time to Clinical Improvement in Patients With Severe and Life-threatening COVID-19: A Randomized Clinical Trial. *JAMA* **324**, 460-470, doi:10.1001/jama.2020.10044 (2020).

24 AlQahtani, M. *et al.* Randomized controlled trial of convalescent plasma therapy against standard therapy in patients with severe COVID-19 disease. *Sci Rep* **11**, 9927, doi:10.1038/s41598-021-89444-5 (2021).

25 Pouladzadeh, M. *et al.* A randomized clinical trial evaluating the immunomodulatory effect of convalescent plasma on COVID-19-related cytokine storm. *Intern Emerg Med* **16**, 2181-2191, doi:10.1007/s11739-021-02734-8 (2021).
